# Electromechanical control of nitrogen-vacancy defect emission using graphene NEMS

**DOI:** 10.1038/ncomms10218

**Published:** 2016-01-08

**Authors:** Antoine Reserbat-Plantey, Kevin G. Schädler, Louis Gaudreau, Gabriele Navickaite, Johannes Güttinger, Darrick Chang, Costanza Toninelli, Adrian Bachtold, Frank H. L. Koppens

**Affiliations:** 1 ICFO-Institut de Ciencies Fotoniques, The Barcelona Institute of Science and Technology, Castelldefels, Barcelona 08860, Spain; 2 CNR-INO, Istituto Nazionale di Ottica, LENS Via Carrara 1, Sesto Fiorentino (FI) 50019, Italy; 3 ICREA – Institució Catalana de Recerça i Estudis Avancats, Barcelona, Spain

## Abstract

Despite recent progress in nano-optomechanics, active control of optical fields at the nanoscale has not been achieved with an on-chip nano-electromechanical system (NEMS) thus far. Here we present a new type of hybrid system, consisting of an on-chip graphene NEMS suspended a few tens of nanometres above nitrogen-vacancy centres (NVCs), which are stable single-photon emitters embedded in nanodiamonds. Electromechanical control of the photons emitted by the NVC is provided by electrostatic tuning of the graphene NEMS position, which is transduced to a modulation of NVC emission intensity. The optomechanical coupling between the graphene displacement and the NVC emission is based on near-field dipole–dipole interaction. This class of optomechanical coupling increases strongly for smaller distances, making it suitable for nanoscale devices. These achievements hold promise for selective control of emitter arrays on-chip, optical spectroscopy of individual nano-objects, integrated optomechanical information processing and open new avenues towards quantum optomechanics.

## Abstract (web summary)

Active control of optical fields at the nanoscale is difficult to achieve. Here, the authors fabricate an on-chip graphene NEMS suspended a few tens of nanometres above nitrogen vacancy centres and demonstrate electromechanical control of the photons emitted by electrostatic tuning of the graphene NEMS position.

## Graphical abstract

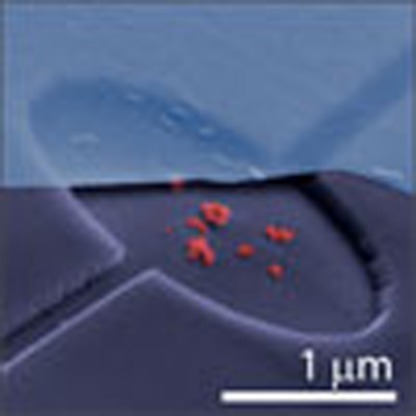

Active, *in situ* control of light at the nanoscale remains a challenge in modern physics and in nanophotonics in particular^1^,^2^,^3^. A promising approach is to take advantage of the technological maturity of nanoelectromechanical systems (NEMS) and to combine it with on-chip optics^4^,^5^,^6^. However, in scaling down the dimensions of such integrated devices, the coupling of a NEMS to optical fields becomes challenging. Despite recent advances in nano-optomechanical coupling^7^,^8^,^9^,^10^, *in situ* control of light at the nanoscale with an on-chip NEMS has not been accomplished thus far. In this context, recent work has shown that graphene is an ideal platform for both nanophotonics^11^,^12^,^13^,^14^,^15^ and nanomechanics^16^,^17^,^18^.

Here we demonstrate a single device, combining these two platforms. In this device, the transduction between nanomotion and an optical field is due to a strong modification of an emitter's relaxation rate and light emission when graphene is placed in its near field^14^,^19^,^20^,^21^,^22^,^23^,^24^, at nanometre-scale distances. The coupling strength increases strongly for shorter distances and is enhanced because of graphene's two-dimensional (2D) character and linear dispersion. As such, this near-field hybrid optomechanical coupling mechanism between graphene and a point dipole is intrinsically nanoscale in comparison with the evanescent coupling involving micron-scale cavities and waveguides in previous lines of work^4^,^25^,^26^,^27^. In addition, owing to its electromechanical properties, graphene NEMS can be actuated and deflected electrostatically over few tens of nanometres with modest voltages applied to a gate electrode^17^,^28^,^29^,^30^,^31^. The graphene motion can thus be used to modulate the light emission, while the emitted field can be used as a universal probe^19^,^20^,^23^ of the graphene position.

The coupling between an emitter and graphene can manifest itself in various ways, such as Stark shift^32^, dipolar-coupling-induced modification of the emission intensity^20^,^21^,^22^,^33^ or energy (Casimir–Polder^34^) or as energy transfer to graphene plasmons^11^,^14^. In our experiment, we use the dipole–graphene coupling to control the nitrogen-vacancy centre (NVC) emission by the graphene displacement. This effect is due to non-radiative energy transfer (n-RET) and is mediated by dipolar interactions between the emitting point dipole and induced (lossy) dipoles in graphene, as shown schematically in Fig. 1a. As a consequence, it gives rise to a diverging decay rate^19^ *Γ*_G_∝*d*^−4^ of the emitter in the presence of graphene at a separation *d*=5–50 nm. Therefore, the emission is reduced with decreasing graphene-emitter separation. Here graphene offers the advantage to be a 2D gapless broadband energy sink. First, the distance dependence of the energy transfer rate is governed by material-free parameters and wavelength, which can be exploited as a universal nanoruler^19^,^20^,^23^. Second, the enhanced dipolar coupling strength and stronger distance dependence (*d*^−4^ compared with *d*^−3^ for bulk materials^20^) makes the near-field dipolar interaction a more effective and divergent coupling mechanism between a graphene NEMS and a fluorescent emitter.

## Results

### Hybrid graphene–NVC optomechanical device

**Figure 1:**
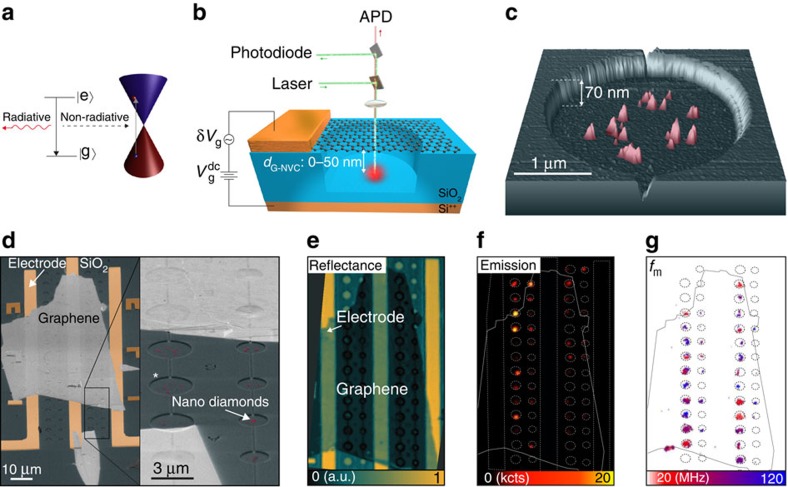
Graphene–NVC hybrid optomechanical device. (**a**) Energy diagrams of an optical emitter and graphene at the K point of the Brillouin zone (Dirac cone). For small separations *d*_G–NVC_<50 nm, the relaxation of the excited emitter is predominantly due to near-field dipole–dipole interaction by excitation of electron–hole pairs in graphene. (**b**) Sketch of the hybrid optomechanical device. The graphene resonator is driven and displaced electrostatically by d.c. and a.c. voltages *V*_g_^dc^ and *δV*_g_, while its nanomotion is measured optically via the emitter using single-photon counters (APD) and by interferometry. (**c**) False colour AFM topology of nanodiamonds (red) deposited in the centre of a hole etched into SiO_2_. (**d**) False colour scanning electronic micrograph (SEM) of arrays of hybrid graphene devices. The labelled hole corresponds to **c**. Graphene is closely suspended over the nanodiamonds (0–50 nm) and clamped at the edges of the holes by Vander Waals interactions. (**e**) False colour confocal reflection map of the same device shown in **c** at *T*=3 K. At each laser position, both emission and a mechanical spectrum are recorded, thus providing a spatial map of the NVC emission (**f**), and the extracted mechanical resonance frequency *f*_m_ (**g**).

To harness near-field dipolar interactions for nano-optomechanical coupling, we propose and demonstrate a novel type of integrated hybrid device as shown in Fig. 1b. Our device consists of a four-layer graphene membrane designed to be suspended some 10–50 nm above a nanodiamond containing one or a few fluorescent NVCs^35^,^36^, as shown in Fig. 1c,d. By applying a combination of a d.c. and a.c. voltage to the conducting graphene membrane relative to the doped silicon backgate, we control the graphene–NVC separation and can simultaneously drive the resonator at radio frequencies. Thus, our device enables electromechanical control of the NVC emission, and complementarily this emission is a transducer of the resonator's nanomotion.

Our hybrid devices are fabricated in arrays (10–100 devices per chip); however, we address them individually. The NVC fluorescence is monitored by a custom-made scanning confocal microscope (Fig. 1f), which simultaneously records the reflectance (Fig. 1e) from the device. These reflection measurements allow us to detect the nanomotion of the graphene resonators by interferometry (*cf.* Supplementary Notes 1, 2 and Supplementary Fig. 1), yielding a map of the mechanical resonance frequency *f*_m_ as shown in Fig. 1g. Here *f*_m_ depends on drum diameter and can vary between similar drums because of inhomogeneous strain and the presence of ripples as well as photothermal effects^37^. Together, Fig. 1f,g reveal the colocalization of the emitters and graphene resonators by optical measurements.

### Electromechanical control of NVC emission

To quantitatively study and control the near-field interaction between the NVCs and the graphene resonator, we tune the graphene-emitter separation electrostatically. The membrane is attracted towards the NVCs by applying a potential difference 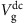 between the backgate and the graphene drum. Optical interferometry measurements (*cf.* Supplementary Note 2 and Supplementary Fig. 2) show that the static deflection of the graphene scales as 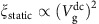, in agreement with electrostatic actuation. These measurements allow actuation calibration on the order of 1.2±0.1 nm V^−2^ for the sample shown here (*cf.* Supplementary Note 3). For a given value of 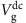, the graphene-emitter separation is 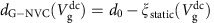, where *d*_0_ is the initial graphene-emitter separation extracted from the measured device topology (Fig. 1c). Such an electrostatic actuation provides *in situ* and stable control of the graphene deflection from its initial position to the point of contact with the nanodiamond (*cf.* Supplementary Fig. 3).

**Figure 2:**
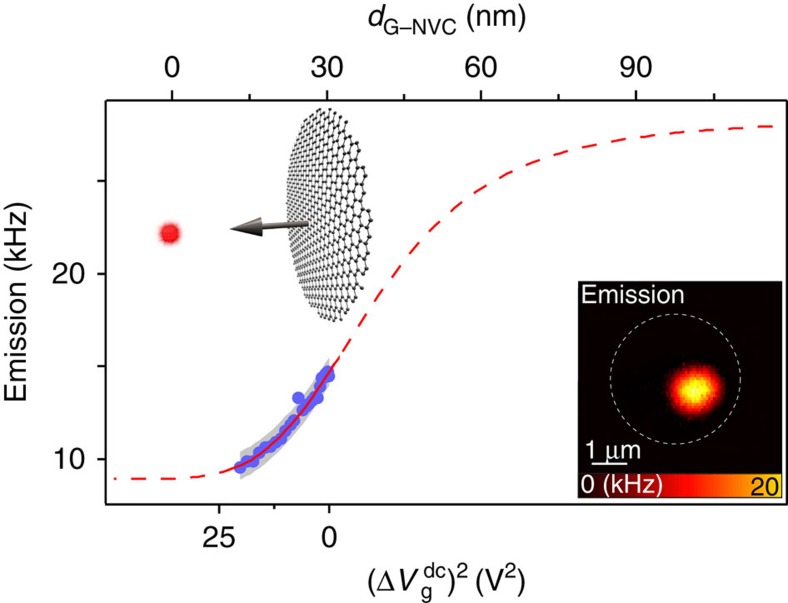
*In situ* electromechanical control of dipolar coupling. Dependence of measured NVC emission intensity (blue) on the graphene-emitter separation *d*_G*–*NVC_, controlled by electrostatic deflection of the membrane. With increasing 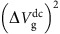, the membrane approaches the NVC, thereby reducing *d*_G–NVC_ and quenching the NVC emission. Data can be fitted with an n-RET model (red), which also allows deflection calibration. For large separations, the extrapolated emission rate agrees with measured emission of nanodiamonds in the absence of graphene. The grey band is an uncertainty interval of width 1,500 Hz, estimated in detail in the Supplementary Note 6. Inset: optical emission map of the hybrid system showing localized NVC emission (dashed line: graphene resonator outline).

We use this *in situ* control to extract the separation between the graphene and a localized emitter, a quantity that is difficult to extract using far-field or local probe techniques. Furthermore, we experimentally verify that the graphene-emitter coupling is due to n-RET by measuring the NVC emission as a function of the membrane position. As shown in Fig. 2, we observe a nonlinear reduction of the NVC emission as the membrane is electrostatically deflected towards the nanodiamond. As introduced above, the emitter decay rate *Γ*_G_(*d*) has a nonlinear separation dependence, which induces a separation-dependent total emission strength given as^20^ (black line in Fig. 2):


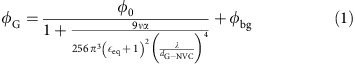


Here, *φ*_0_ is the emission of a single emitter in the absence of graphene, *ν*∈[1,2] takes into account the emitting dipole orientation, *α* is the fine structure constant, 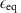 is the equivalent relative permittivity of the separating medium (*cf.* Supplementary Note 2) and *λ* is the emission wavelength, which we take to be the peak wavelength of the NVC emission spectrum (*cf.* Supplementary Note 4 and Supplementary Fig. 4). We fit the data with the emission model shown above where we consider energy transfer from an individual NVC to graphene^19^. We consider this interacting emitter to be the NVC closest to the graphene, within a nanodiamond containing an ensemble of one to four NVCs^36^. Further, we assume NVCs embedded deeper within the nanodiamond to contribute to a fluorescence background *φ*_bg_ (visible in Fig. 2 for small separations), with negligible dependence on the graphene membrane position (*cf.* Supplementary Note 5 and Supplementary Fig. 5). This background fluorescence inhibits the use of radiative lifetime decay measurements as an alternative method to probe the n-RET separation dependence. However, good agreement of our data with the n-RET model shows that emission measurements provide an indirect optical probe of the graphene position. Given the assumptions described, the main contributions to the measurement uncertainty—shown in Fig. 2 as a grey band—are the broad emission spectrum as well as the uncertainty in the initial membrane position (*cf.* Supplementary Note 6). We remark that the observed emission reduction cannot be a result of an interferometric modulation of the excitation intensity because of graphene deflection. Indeed, for our device geometry (oxide thickness and hole depth) the interferometric effect would lead to an increase in the emission on decreasing *d*_G–NVC_, in contrast to the measurements shown in Fig. 2 (*cf.* Supplementary Note 4 and Supplementary Fig. 3 for further discussion).

### Nanomotion transduction to NVC emission

**Figure 3:**
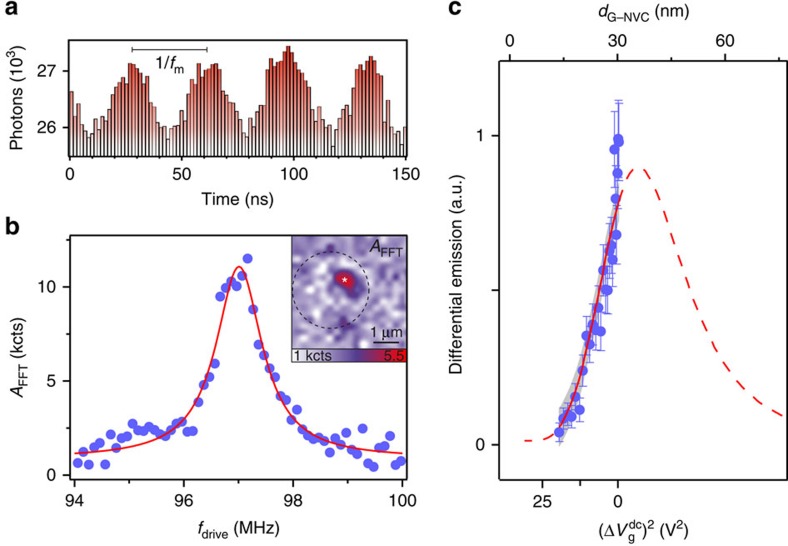
Graphene nanomotion transduction to NVC emission. (**a**) Time trace of NVC emission (bars) modulated by a driven graphene membrane oscillating at *f*_m_ in its near field. Distance-dependent dipolar emitter–graphene coupling imprints the nanomotion of the graphene membrane on the emission. (**b**) Mechanical resonance obtained from differential oscillating NVC emission. Each point corresponds to the amplitude of the Fourier component at *f*=*f*_drive_ of the emission time trace (as in **a**). A Lorentzian fit of the data (red line) yields the same value for *f*_m_ as obtained independently by optical interferometry. Inset: spatial map of Fourier component at *f*=*f*_drive_, localized around the emitter position (*). The dashed circle indicates the graphene drum. (**c**) Dependence of measured (blue) and extrapolated (red) differential NVC emission intensity on the graphene-emitter separation *d*_G–NVC_, normalized by the membrane's resonant oscillation amplitude *A*_m_ (which increases with *V*_g_, thus reducing the measurement uncertainty for small separations *d*_G–NVC_). The grey band is an uncertainty interval (*cf.* Supplementary Note 6).

Our hybrid device enables high frequency and local control of individual emitters at subwavelength scales. To demonstrate this concept, we show optomechanical transduction of radiofrequency graphene resonator nanomotion to NVC emission, by performing time-resolved emission measurements. During a mechanical oscillation cycle, the graphene-emitter separation *d*_G–NVC_ periodically varies with an amplitude *δz*, and the graphene position is imprinted on the NVC fluorescence. To observe this, we drive the resonator capacitively at frequency *f*_drive_ and simultaneously perform time-correlated single-photon counting of the emitted photons over a few mechanical periods. By repeating such synchronized acquisition, we obtain a histogram of photon arrival times modulated at *f*_drive_, as shown in Fig. 3a. As such, this transduction mechanism involves three successive steps: (i) the initial electromechanical actuation of the membrane, followed by (ii) a quasi-instantaneous optomechanical transduction due to n-RET (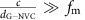, c being the speed of light) and finally (iii) a conversion into a time-resolved electronic signal at the single-photon-counting module. Additional interferometric measurements enable calibration of the driven oscillation amplitude to be ∼1 nm at resonance (*cf.* Supplementary Note 3 and Supplementary Fig. 4). The optomechanical transduction step is linear for *δz*<<*d*_G–NVC_, resulting in the observed sinusoidal emission modulation despite the nonlinear dependence of emission on *d*_G–NVC_.

To reveal the mechanical spectrum of the graphene resonator in the NVC emission, we extract the modulation depth *A*_FFT_(*f*_drive_) defined as the Fourier component of the emission time traces at different frequencies *f*_drive_. At mechanical resonance, both the amplitude of motion *δz*(*f*_m_) and *A*_FFT_(*f*_m_) are greatest. Indeed, by sweeping *f*_drive_ through *f*_m_ (independently measured by interferometry), we can reconstruct the mechanical spectrum of the graphene resonator (Fig. 3b) through the near-field transduction mechanism.

This transduction strength can be tuned *in situ*. To this end, we record *A*_FFT_(*f*_m_) while varying the stationary separation *d*_G–NVC_, as shown in Fig. 3c. Here the differential emission Δ*A*_FFT_=*A*_FFT_(*f*_m_)/*A*_r_(*f*_m_) is the measured emission modulation amplitude *A*_FFT_(*f*_m_), normalized to the resonant oscillation amplitude *A*_r_(*f*_m_) as obtained from interferometry. This normalization is necessary to compensate the increase in *A*_r_ with increasing backgate voltage as 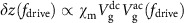, where *χ*_m_ is the mechanical susceptibility. The measurements shown in Fig. 3c reveal that Δ*A*_FFT_ diminishes with increasing 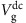. Indeed, while the near-field interaction diverges with decreasing *d*_G–NVC_, the observed emission, and thus the transduced signal, is quenched. Our data can be fitted by the derivative of NVC emission with respect to *d*_G–NVC_, as expected from equation (1). We find that the largest transduction would be obtained for a separation of 35±3 nm. These results, summarized in Fig. 3c, show that we achieve active control of the optomechanical coupling strength by tuning the separation between a local emitter and a vibrating graphene NEMS.

Finally, we explore the localized nature of the near-field coupling mechanism in the normal plane through time-resolved emission from our hybrid device over the whole area of the driven resonator. A spatial map of *A*_FFT_(*f*_m_) (inset of Fig. 3b) shows a stronger signal at the NVC site, as expected for such n-RET coupling between graphene and a point-like emitter. This observation highlights the intrinsic localization of the interaction, confined within a subwavelength volume. As such, a small ensemble of emitters can be used as a local transducer of the graphene NEMS motion, which enables eigenmode shape reconstruction. On the other hand, driving the NEMS at higher-order mechanical modes, with subwavelength spatial modulations, would allow addressing and coupling to individual emitters distributed over separations unresolvable with far-field optics.

## Discussion

Our device also holds promise for dissipative optomechanics experiments. To quantify the dissipative coupling demonstrated here, we use an established formalism^38^,^39^ to extract a dimensionless dissipative coupling strength 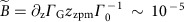 and the dissipative optomechanical coupling rate *∂*_z_*Γ*_G_=6 MHz nm^−1^, where *Γ*_G_ is the decay rate of the emitter in presence of graphene, *z*_zpm_∼65 fm is the zero-point motion of the resonato and, *Γ*_0_∼30 MHz is the intrinsic decay rate of an NVC in a nanodiamond^40^ (*cf.* Supplementary Note 7). This compares favourably with existing dissipative optomechanical devices such as a microdisk coupled to an optical waveguide^41^, a graphene resonator coupled to a microsphere optical resonator^25^ or a photonic crystal nanocavity^42^. The dissipative coupling quantified here takes into account spectral broadening of the emitter we selected. We remark that Fourier limited linewidth emitters such as molecules^43^ or NVC's in bulk diamonds^44^, in nanocrystals or implanted close to a surface^45^ are readily available.

In conclusion, we have realized a novel device comprising a graphene NEMS dissipatively coupled to NVCs by near-field dipolar coupling. Our work offers interesting perspectives for lock-in detection of weak fluorescence signals, NEMS position sensing, electromechanical control of emitters on-chip and fast electromechanical light modulation at the single-photon level. On the basis of the system presented, we envision a similar device that harnesses vacuum forces^34^, inducing a divergent and dispersive optomechanical coupling between a quantum emitter and a 2D system. For instance, using a semiconducting 2D resonator (for example, MoS_2_) instead of graphene allows to supress the n-RET dissipative coupling, so that the coupling is purely dispersive. A hybrid system combining such resonators (*f*_m_∼10–100 MHz) placed at 20–40 nm from a lifetime-limited linewidth (*Γ*_0_/2*π*∼50 MHz) emitter, such as a dibenzoterrylene (DBT) molecule^43^, would feature a large dispersive coupling strength^34^ of *g*_0_/2*π*∼10–100 MHz. Such systems may approach the ultrastrong coupling regime (*g*_0_/2*π*>*f*_m_, *Γ*_0_/2*π*). Importantly, as discussed in ref. 34, this type of system may enable significant squeezing of mechanical motion at room temperature and position detection on timescales that are short compared with 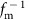 because of its extremely large displacement sensitivity. This would then enable coherent manipulation of mechanical and optical degrees of freedom at the level of single quantum^7^,^46^,^47^.

## Methods

### Fabrication

Initially, we pattern electrodes on the surface of a 285-nm-thick SiO_2_ layer thermally grown on a p-doped Si chip using electron beam lithography (EBL) and thermal evaporation of Au. Holes of 80–100 nm depth and with diameters ranging from 2 to 5 μm are patterned using EBL and subsequent reactive ion etching. Air escape trenches of 150 nm width are etched between adjacent holes to avoid buckling of the subsequently transferred graphene membrane when the device is placed in vacuum. Using a mask exposed by EBL, we deposit nanodiamonds selectively within the etched holes by spin-drop casting^48^. We use a commercially available suspension of ∼40 nm diameter nanodiamonds, which contain one to four embedded NVCs. Graphite is exfoliated mechanically using commercial polydimethylsiloxane (PDMS) sheets. Large, few-layer graphene flakes on the PDMS are identified under an optical microscope by contrast measurement. Identified flakes (*cf.* Supplementary Note 1 and Supplementary Fig. 1) are then transferred to target hole arrays using a three-axis micromanipulator^49^, forming graphene membranes closely suspended above nanodiamonds. This dry stamp approach has the advantage of high spatial transfer precision and avoids contamination by process liquids.

### Electrical device actuation and optical readout

Measurements are performed under vacuum in a cryostat. Data shown in Fig. 1e–g are taken at 3 K and data shown in Figs 2 and 3 are taken at 300 K at 10^−6 ^mbar. Our hybrid devices are actuated electrically using a waveform generator and a low-noise voltage source to provide a.c. and d.c. voltage signals. We use a custom-built confocal microscope to locally illuminate the device with 532-nm laser light and simultaneously readout both the reflected and emitted light. The reflection signal is detected using a fast photodiode for the high-frequency reflection component and a powermeter for the static reflection component. We read out the high-frequency photodiode signal with a lock-in amplifier or spectrum analyser to study the resonator mechanics. The emitted light is detected by an avalanche photodetector (APD) combined with 532-nm notch and dichroic filters. Time-resolved emission traces are recorded by feeding the APD signal to a time-correlated single-photon counter, triggered at the radiofrequency driving voltage provided by the waveform generator. The electrostatic actuation is kept low (typically, 1–10 μV) to remain in the linear mechanical regime^18^. Data shown in Fig. 1f are obtained by interferometric detection of the electrostatically driven motion of graphene drums (*cf.* Supplementary Note 2). Graphene motion is detected as a modulation of the reflected light. The drums are driven at a frequency *f*, and the optical reflection modulation amplitude *A*(*f*) at this frequency is read out via a lock-in type of measurement. For each position of the laser, we fit the recorded spectra and extract relevant parameters (*f*_m_, *Q*, amplitude). For most resonators, we do not observe higher-mode resonances within our measurement frequency range, for which reason we attribute our extracted peak frequency to be the mechanical resonance of the fundamental mechanical mode.

### Extraction of dissipative optomechanical coupling strength

The detection sensitivity of our readout scheme is ∂_z_*φ*=∂*φ*/∂*Γ*_G_.∂_z_*Γ*_G_, where *φ* is the number of emitted photons per second. From Fig. 2, one can extract ∂_z_*φ*=300 Hz nm^−1^ for a separation of 30 nm and then evaluate |∂*φ*/∂*Γ*_G_|=5 × 10^−5^ using a background-corrected expression for *φ*. Together with the experimental value for ∂_z_*φ*, this yields ∂_z_*Γ*_G_=6 × 10^6^Hz nm^−1^. Given a zero-point motion amplitude *z*_ZPM_=65 fm, the dimensionless dissipative coupling strength 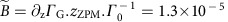 is extracted following the definition of Elste *et al*.^38^ and Weiss *et al*.^39^. See Supplementary Note 7 for more details.

## Additional information

**How to cite this article:** Reserbat-Plantey, A. *et al*. Electromechanical control of nitrogen-vacancy defect emission using graphene NEMS. *Nat. Commun.* 7:10218 doi: 10.1038/ncomms10218 (2016).

## Supplementary Material

Supplementary Information Supplementary Figures 1-5, Supplementary Notes 1-7 and Supplementary References.
