# Supplementary material for: Electromechanical control of nitrogen-vacancy defect emission using graphene NEMS
Source: Nat Commun. 2016 Jan 8;7:10218. doi: 10.1038/ncomms10218 (PMC4729859; doi:10.1038/ncomms10218)
Supplement: Supplementary Information — Supplementary Figures 1-5, Supplementary Notes 1-7 and Supplementary References. [file ncomms10218-s1.pdf]

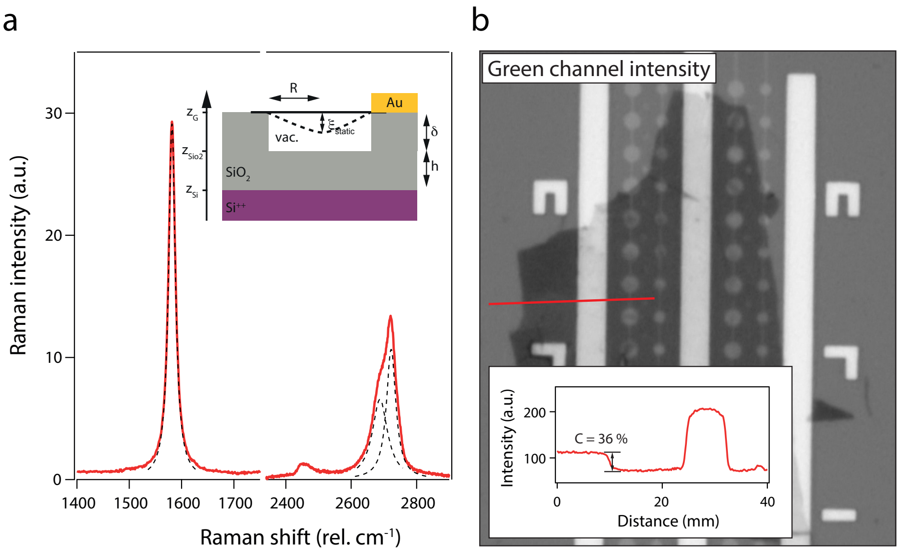

Supplementary Figure 1. **a) Graphene Flake Characterization.** Raman spectrum of the graphene flake used in our device. **Inset: Simplified device model.** We consider an electrically contacted graphene membrane of radius R located at z_G and suspended in vacuum at a distance δ over a layer of SiO_2 (thickness h). When the device is illuminated by laser radiation incident along the z-axis, an interference pattern is formed by reflection at the material interfaces located at z_{Si} and z_{SiO_2}. The graphene membrane absorbs a position-dependent fraction of the field intensity and can be electrostatically deflected through the interference pattern towards the Si^{++} backgate by a distance ξ_{static}. **b** Green channel intensity of an optical micrograph of the multilayer graphene flake used in our device. A line section (inset) reveals a contrast of $C \approx 36\%$ between the graphene-covered area and its surroundings.

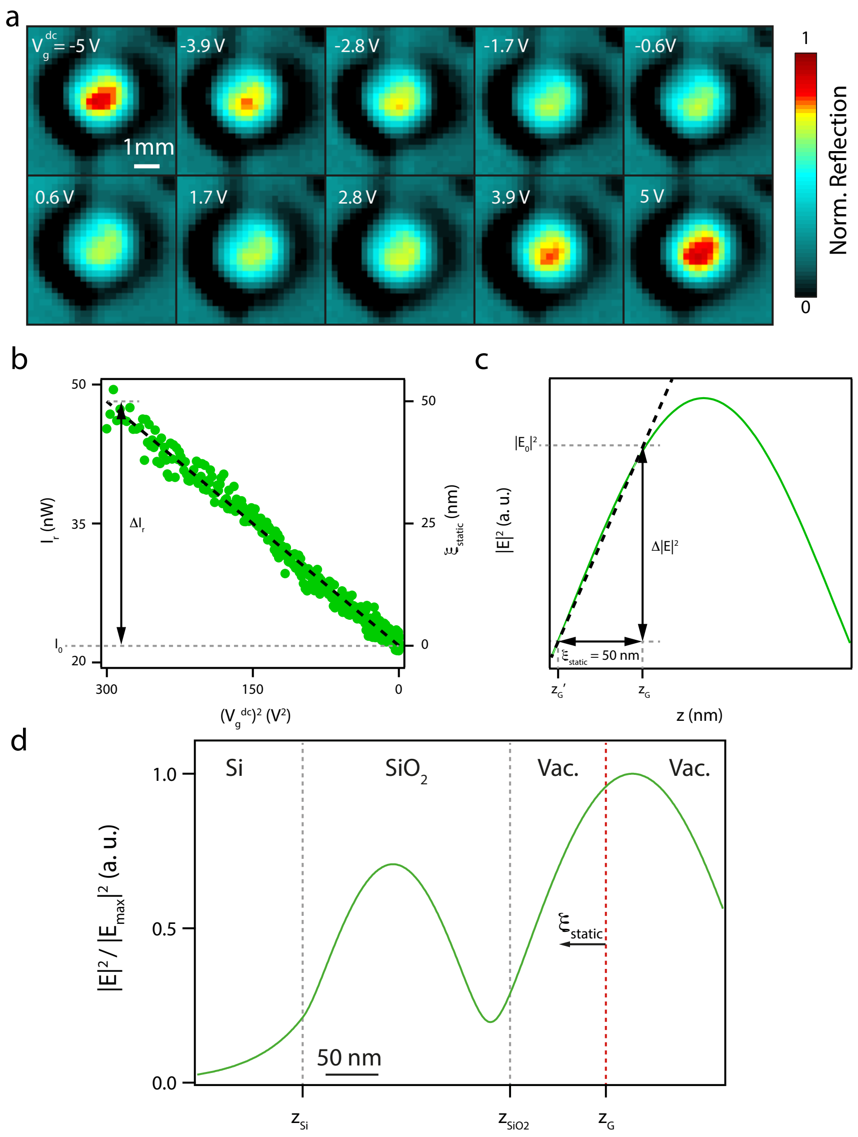

Supplementary Figure 2. **a) Reflected intensity as a signature of graphene drum deflection.** Spatial maps of reflected intensity $I_r(x, y)$ from an electrostatically graphene drum for a range of applied backgate voltages $V_g^{dc} \in [-5, 5]V$. **b) Static deflection calibration by reflection measurement.** Reflected intensity variation with applied V_g^{dc}. The black dashed line shows a linear fit of the data. **c) Static deflection calibration by reflection measurement.** Model of $|E(z)|^2$ for device measured in **b**. A graphene membrane initially at position z_G is deflected by a distance ξ_{static} to a final position z'_G. By comparison of the model $\frac{\Delta|E|^2}{|E(z_0)|^2}$ and the measurement $\frac{\Delta I_r}{I_0}$, we extract an estimate for calibration, indicated as the right axis in **b**.**d) Calculated electric field intensity.** $|E(z)|^2$ in the Si, SiO_2 and vacuum regions of the device (interfaces indicated by dashed grey lines). $|E(z)|^2$ is normalised to the maximum field intensity $|E_{max}|^2$, and the graphene membrane is located at z_G (red dashed line). Here, $\delta = 90$ nm.

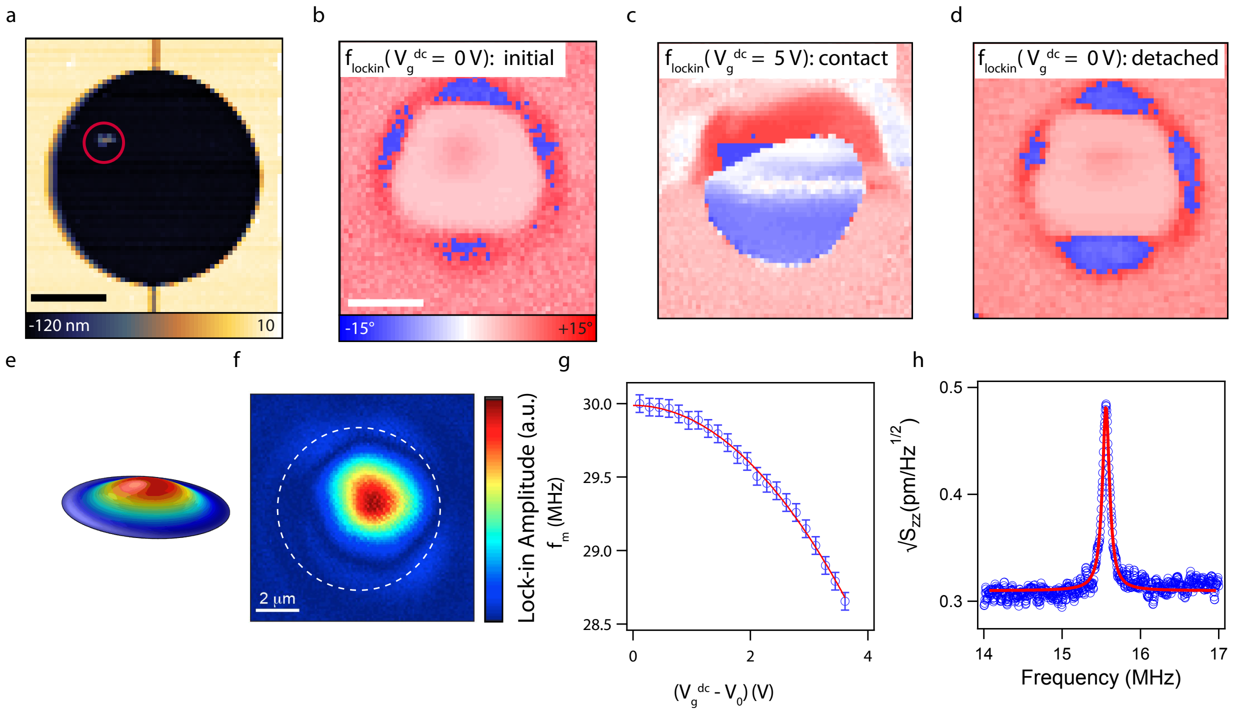

Supplementary Figure 3. **a) Contact and detachment of graphene membrane with a nano-diamond.** AFM map of sample depth before graphene transfer. The nano-diamond position is indicated within the red circle. **b,c,d)** Maps of lock-in amplifier phase ϕ_{lockin} over the area of a resonantly driven graphene drum, indicating the mechanical phase of the resonator for different deflections. Initially in its undeflected state at $V_g^{dc} = 0$ V (**b**), the drum is deflected to be in partial contact with the nano-diamond at $V_g^{dc} = 5$ V (**c**) after which it can be detached from the nano-diamond and brought back to its initial state at $V_g^{dc} = 0$ V again (**d**). **e) Mode profiles of a circular graphene drum resonator.** Finite-element simulation of the fundamental mode profile and **f**) experimentally measured mode profile. The dashed line indicates the graphene membrane perimeter. **g) Electrostatic tuning of graphene's mechanical properties.** Tuning of graphene's mechanical frequency f_m with backgate voltage V_g^{dc} due to electrostatic softening. The red dashed line is a fit of the data as described in the text. **h) Calibrated, single-sided power spectral density of a thermally driven graphene drum.** A Lorentzian fit of the data yields a mechanical resonance frequency $f_m = 15.5$ MHz and a mechanical quality factor $Q = 156$ at $T = 300K$. Scale bars are 1 μm.

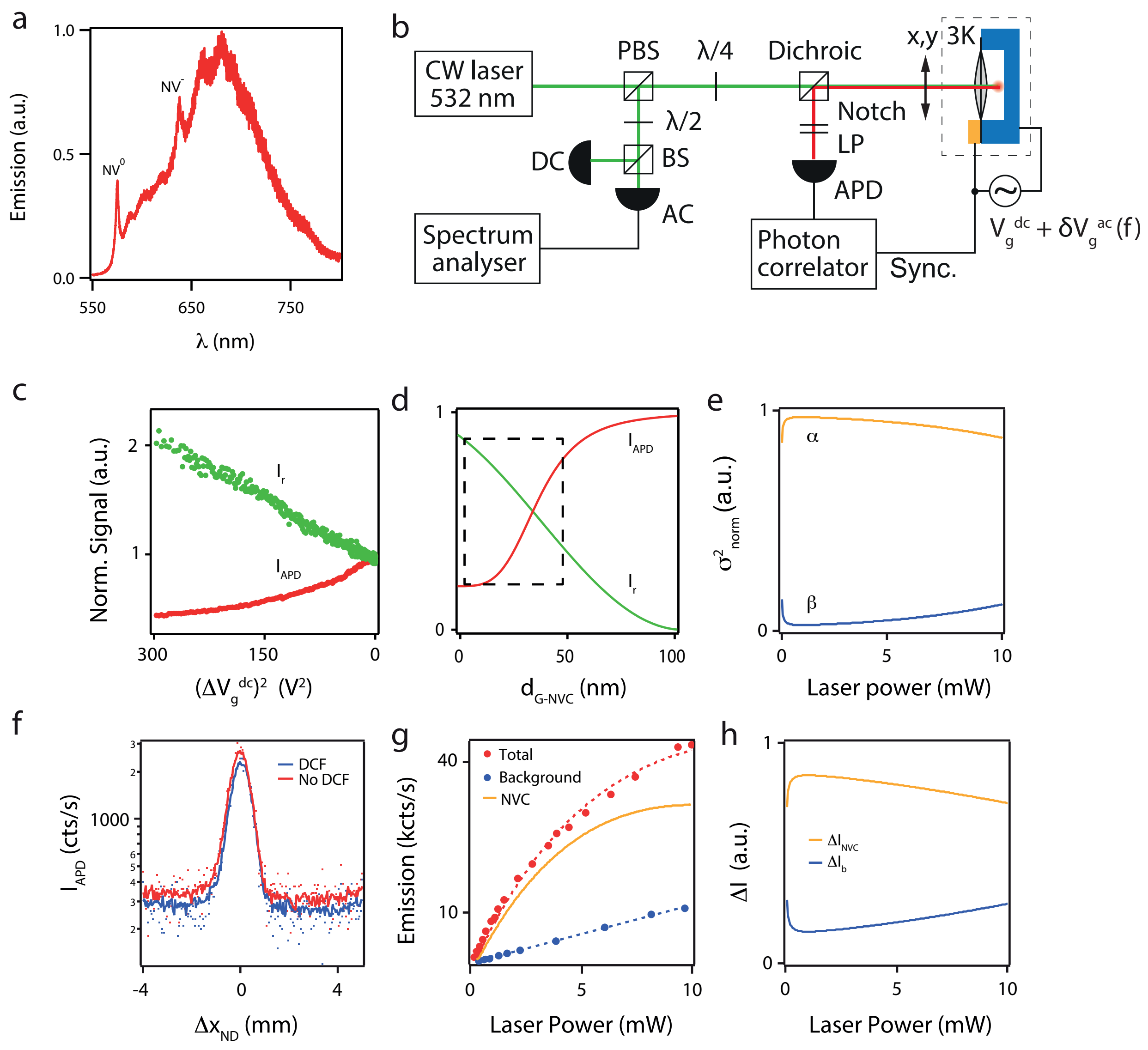

Supplementary Figure 4. **a)** NVC emission spectrum from nano-diamonds at 3 K. **b**Scanning confocal microscope setup. **c** Measured reflection I_r (green) and emission I_{APD} (red) with electrostatic deflection, normalised to initial signal level at $\Delta V_g^{dc} = 0$. **d)** Model of I_r and I_{APD} against emitter-graphene separation d_{G-NVC}. The area enclosed by the dashed line indicates the region explored by the deflected graphene in **c)** and shows the same behaviour. **e)** Normalised variance of NVC emission α and background signal β against excitation laser power. At approx. 1 mW, α constitutes 96% of the normalised total signal variance.**f)** Line traces of measured optical signal I_{APD} in the sample plane around a nano-diamond site at $\Delta x_{ND} = 0$, with a dichroic filter (DCF) in place (blue). Removing the DCF gives rise to a net increase of I_{APD} by 16% (red).**g)** Excitation laser power dependence of I_{APD} (red), I_b (blue) and extracted NVC emission strength $I_{NVC} = I_{APD} - I_b$ (yellow), showing saturation. **h)** Excitation laser power dependence of relative contribution of NVC emission (yellow) and background (blue) to the measured optical signal.

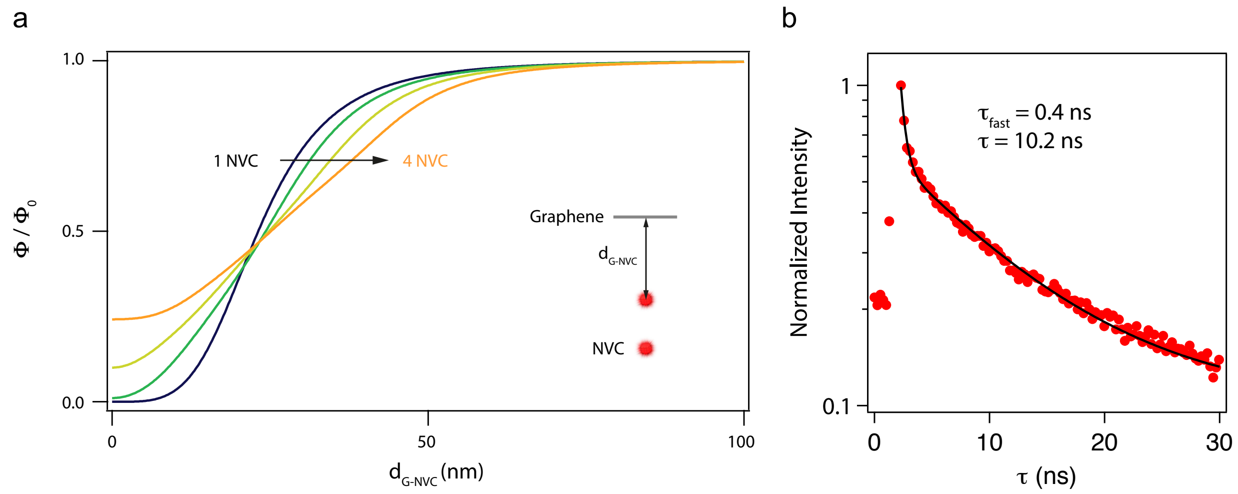

Supplementary Figure 5. **a) Model of emission quenching behaviour of NVC ensembles.** We model an ensemble of 1-4 NVCs, spaced by 15 nm within a nano-diamond. The distance d_{G-NVC} describes the distance of graphene to the closest NVC (see inset). As the number of emitting NVCs increases, the overall quenching effect is reduced as the relative proportion of NVCs interacting with graphene is reduced.**b) Lifetime measurement of NVCs in nano-diamond.** Data show bi-exponential behavior (τ_{fast}, τ), as previously reported[3,14] for NVCs in nano-diamonds.

Supplementary Note 1. DETERMINATION OF GRAPHENE FLAKE THICKNESS

We record a Raman spectrum (pump laser 532 nm) of the graphene flake in our device to determine its thickness (Supplementary Figure 1 a). The 2D band profile clearly shows the presence of more than one peak, ruling out the presence of single layer graphene. Even if it is generally accepted that the Raman response of few layer graphene becomes almost indistinguishable for samples with more than five layers, the shape and peak position is consistent with four layer graphene, as discussed for instance by Ferrari et al.[7]. Indeed, the asymmetry of $2D_1$ and $2D_2$ peak intensity is not as pronounced as it is for a graphite sample, for instance. In addition, optical contrast measurements support that the graphene flake indeed consists of four layers. Supplementary Figure 1 b shows the green channel intensity of reflected light from our device under white light illumination, obtained by extraction of the green RGB channel from an optical micrograph. A line section through shows a contrast $C = \frac{(I_0 - I_{graphene})}{I_0} \approx 36\%$ between the uncovered (I_0) and graphene-covered ($I_{graphene}$) areas. From the literature value of 8-10 % contrast for a single layer of graphene on 285 nm of SiO2 thickness[2], we therefore deduce the flake thickness to be 4 layers.

Supplementary Note 2. MEMBRANE ACTUATION AND DETECTION

Understanding the modulated reflection signal resulting from the motion of the graphene membrane in our device device (shown schematically in Supplementary Figure 1) requires a model of the interference pattern in which the membrane moves. We employ a transfer matrix model for the optical reflection and transmission of multilayer stacks as detailed by Burkhard et al.[4] to calculate the electric field distribution $E(z)$ within our device.

Supplementary Figure 2-cd shows the resulting field intensity $|E(z)|^2$ obtained from this model for normally incident laser radiation at $\lambda = 532$ nm on a typical sample geometry with a hole depth of $\delta = 120$ nm, resulting in $h = 165$ nm for 285 nm SiO_2 thickness. The position z_G of an ideally suspended graphene drum at the surface of SiO_2 (*i. e.* without slack) is also indicated.

We consider suspended graphene as an absorber with absorption coefficient $\pi\alpha \approx 2.3\%$ per layer (valid for up to five layers of graphene surrounded by vacuum[12]). Hence, the presence

of graphene modifies the intensity of the optical field but not its phase. The fraction of light absorbed by graphene is proportional to the electric field intensity at its position $|E(z_G)|^2$.

$$\frac{I_r(z_G)}{I_0} = 1 - n_g \pi \alpha \frac{|E(z_G)|^2}{|E_{max}|^2} \tag{1}$$

where I_0 is the measured reflection intensity in the absence of graphene, n_g the number of graphene layers and $|E_{max}|^2$ the maximum field intensity. From Eq. 1 we see that the net reflected intensity is minimum when graphene is located at the position of $|E_{max}|^2$.

When the graphene membrane is displaced from its initial position z_G by a distance ξ_{static} towards the backgate, the resulting relative change in the reflected intensity is equal to the relative change of the field intensity due to the linearity of Eq. 1:

$$\frac{I_r(z_G - \xi) - I_r(z_G)}{I_r(z_G)} = \frac{|E(z_G - \xi)|^2 - |E(z_G)|^2}{|E(z_G)|^2} \tag{2}$$

This relation allows us to infer the displacement ξ_{static} from intensity measurements using a model for the electric field intensity of our device.

In the following, we derive the expressions for the electrostatic deflection of a circular graphene drumhead. Here, we adapt this problem to our experimental situation with similar notation to that used by Weber *et al.*[15] (see also Katsnelson[8] and Landau[9]). When the graphene membrane is under tension and subjected to an external electrostatic pressure $P(x, y)$ along the z direction, its deformation $\xi(x, y, t)$ obeys the equilibrium condition given by :

$$\rho_{2D} \frac{\partial^2 \xi}{\partial t^2} = T \nabla^2 \xi + P(x, y) \tag{3}$$

where $\rho_{2D} = n_g \rho_g \eta$ is the graphene sheet mass density which depends on the number of graphene layers n_g, graphene's intrinsic mass density ρ_g and a correction factor $\eta \geq 1$ to take into account the adsorbates on the membrane. $T = Et\epsilon n_g$ is the stretching force per unit length at the edge of the membrane, which depends on the Young's modulus $E = 1$ TPa, the thickness (t=0.3 nm) of a graphene layer, the radial strain ϵ and the number n_g of graphene layer considered. By applying a constant voltage V_g^{dc} between the graphene and the gate electrode, we generate an electrostatic force F_{el} given as:

$$F_{el} = \frac{1}{2} \partial_z C_{eq} \left(V_g^{dc} \right)^2 \tag{4}$$

where C_{eq} is the equivalent gate capacitance of the system which contains two dielectric layers: vacuum and SiO_2. This conservative force arises from an electrical potential $U_{el} =$

$\frac{1}{2}C_{eq}\left(V_g^{dc}\right)^2$. By expansion of the equivalent capacitance, we find:

$$U_{el} = \iint dxdy \frac{\epsilon_0 \left(V_g^{dc}\right)^2}{2}\left[\frac{1}{\delta+\frac{h}{\epsilon_r}} + \frac{\xi}{\left(\delta+\frac{h}{\epsilon_r}\right)^2} + \frac{\xi^2}{\left(\delta+\frac{h}{\epsilon_r}\right)^3} + \frac{\xi^3}{\left(\delta+\frac{h}{\epsilon_r}\right)^4} + O\left(\xi^4\right)\right] \quad (5)$$

The electrostatic pressure introduced in Eq. 3 fulfills the following equation :

$$\begin{aligned} F_{el} = \frac{\partial U_{el}}{\partial z} &= \iint dxdy P(x,y) \\ &= \iint dxdy \frac{\epsilon_0 \left(V_g^{dc}\right)^2}{2\left(\delta+\frac{h}{\epsilon_r}\right)^2}\left[1 + \frac{2\xi}{\delta+\frac{h}{\epsilon_r}} + \frac{3\xi^2}{\left(\delta+\frac{h}{\epsilon_r}\right)^2} + \frac{4\xi^3}{\left(\delta+\frac{h}{\epsilon_r}\right)^3} + O\left(\xi^4\right)\right] \end{aligned} \quad (6)$$

where $\epsilon_{SiO_2} = \epsilon_r\epsilon_0 = 3.9 \cdot 8.85\ 10^{-12}$ F m^{-1} is the dielectric constant of the SiO_2 layer, h is the SiO_2 layer thickness and δ is the distance from the non-deformed graphene drum to the vacuum/SiO_2 interface. Reinserting $P(x,y)$ into Eq. 3 yields an analytical solution for the deformation:

$$\xi(x,y,t) = \xi_{static}(x,y) + \sum_k \xi_k(x,y)e^{-i\omega t} \quad (7)$$

The graphene drum deflection contains a static term ξ_{static} and a time-dependent term representing the radial mechanical modes. In the steady state, this leads to (cf. Eq. 3):

$$T\nabla^2\xi_{static} = \frac{\epsilon_0 \left(V_g^{dc}\right)^2}{2\left(\delta+\frac{h}{\epsilon_r}\right)^2}\left[1 + \frac{2\xi_{static}}{\delta+\frac{h}{\epsilon_r}} + \frac{3\xi_{static}^2}{\left(\delta+\frac{h}{\epsilon_r}\right)^2} + O\left(\xi_{static}^3\right)\right] \quad (8)$$

For small deflections, we can consider $\frac{2\xi_{static}}{\delta+\frac{h}{\epsilon_r}} \ll 1$ (in our system, $\frac{\delta+\frac{h}{\epsilon_r}}{2} \approx 65$ nm). We now integrate $\nabla^2\xi_{static}$ in radial coordinates to obtain the lowest-order solution for ξ:

$$\begin{aligned} \nabla^2\xi_{static}(r) &= \frac{1}{r}\frac{\partial}{\partial r}\left(r\frac{\partial\xi(r)}{\partial r}\right) = \frac{\epsilon_0\left(V_g^{dc}\right)^2}{2\left(\delta+\frac{h}{\epsilon_r}\right)^2} \\ \frac{\partial\xi}{\partial r} &= \frac{\epsilon_0\left(V_g^{dc}\right)^2}{2\left(\delta+\frac{h}{\epsilon_r}\right)^2}\left[\frac{r}{2}+k_0\right] \\ \xi &= \frac{\epsilon_0\left(V_g^{dc}\right)^2}{2\left(\delta+\frac{h}{\epsilon_r}\right)^2}\left[\frac{r^2}{4}+k_0 r + k_1\right] \end{aligned} \quad (9)$$

At the boundaries of the drum, there is no deflection ($\xi_{static}(r=R)=0$), while at the center, the membrane can be considered as flat ($\frac{\partial\xi_{static}(r=0)}{\partial r} = 0$). Thus, $k_1 = 0$ and $k_2 = -\frac{R^2}{4}$. Therefore, the deflection can be written as:

$$\xi_{static}(r) = \frac{\epsilon_0\left(V_g^{dc}\right)^2}{8\left(\delta+\frac{h}{\epsilon_r}\right)^2}\left[r^2 - R^2\right] \quad (10)$$

At the center of the drum, the deflection is denoted ξ_0:

$$\xi_0 = \xi_{static}(r=0) = -\frac{\epsilon_0 R^2 \left(V_g^{dc}\right)^2}{8T\left(\delta + \frac{h}{\epsilon_r}\right)^2} \tag{11}$$

In the following, we describe the calibration necessary to read out the graphene membrane's static position by measurement of I_r. Maps of reflected intensity $I_r(x,y)$ from a membrane at different values of applied static backgate voltage V_g^{dc} are shown in Supplementary Figure 2. These measurements reveal that the membrane's deflection may be observed by measuring I_r. In addition, these maps display symmetry of the deflection in $(V_g^{dc})^2$ as described by Eq. 10.

Supplementary Figure 2-b shows the linear increase of I_r with $(V_g^{dc})^2$ at the centre of a graphene membrane. We normalize I_r by the intensity I_0 reflected from the undeflected drum and obtain an expression which describes our measurements given by $\frac{I_r}{I_0} = 1 + A\left(V_g^{dc}\right)^2$. Here, A is a device-dependent constant which has dimensions of [V^{-2}] and is extracted from a linear fit of the measured data.

We combine Eq. 10 with the expression for $\frac{I_r}{I_0}$ to obtain an expression which allows us to determine the displacement ξ_0 from the reflected intensity:

$$\xi_0 = \kappa\left[\frac{I_r}{I_0} - 1\right] \tag{12}$$

where

$$\kappa = -\frac{\epsilon_0 R^2}{A8T\left(\delta + \frac{h}{\epsilon_r}\right)^2} \tag{13}$$

is the device-dependent calibration constant.

For large deflections over 65 nm, our calibration is overestimated and does not hold as the higher terms in Eq. 8 must to be taken into account. For the second order in ξ, the solution becomes:

$$\xi_{static}(r) = -\frac{\delta + \frac{h}{\epsilon_r}}{2} - C_1 J_0\left(r\sqrt{b}\right) - C_2 Y_0\left(r\sqrt{b}\right) \tag{14}$$

where $b = \frac{\epsilon_0\left(V_g^{dc}\right)^2}{\left(\delta+\frac{h}{\epsilon_r}\right)^3}$, C_1 and C_2 and constants and $J_0(x)$ and $Y_0(x)$ are Bessel functions of the first and second kind, respectively.

For the case of small deflections, we determine the value of the calibration constant β from the model of the normalized electric field intensity. By insertion of measured values of

the sample geometry, this model yields the electric field intensity profile $|E|^2(z)$ in which the graphene membrane lies. To determine β for a given membrane, we assume that the measured change in the normalized reflected intensity $\frac{\Delta I_r}{I_{r,0}}$ corresponds to the same relative change of the normalized electric field intensity described by the model:

$$\frac{\Delta I_r}{I_{r,0}} \equiv \frac{\Delta |E|^2}{|E_0|^2} \tag{15}$$

where $|E_0|^2$ is the electric field intensity at the initial (undeflected) position of the graphene membrane.

This approximation holds for cases where the maximum deflection is smaller than a period of the standing wave $\xi_{max} \ll \lambda/2$ and in the linear region of the electric field intensity away from nodes of $|E|^2(z)$. Employing this calibration, we also extract the deflection with applied backgate voltage to be to be $\frac{\xi_{static}}{(V_g^{dc})^2} = 0.15 \pm 0.05$ nm / V^2 for a typical membrane with 3 μm diameter as shown in Supplementary Figure 2.

This deflection calibration is only valid for an individual membrane as the intrinsic membrane slack varies over the nanoresonator array due to local material inhomogeneities such as ripples resulting from the graphene transfer process. AFM measurements of arrays of suspended membranes with 3-4 μm diameter provide an estimate of 10-20 nm for the slack, which we include in our model. For a given membrane, measurements of deflection-dependent reflected intensity are reproducible, indicating that the slack stays constant and that the membranes are sufficiently clamped by adhesion to the substrate via van der Waals interactions.

The oscillator position spectrum $S_x[\Omega]$ is defined as the Fourier transform of the temporal autocorrelation function $C_x(\tau) =< x(t)x(t+\tau) >$: $S_x[\Omega] = \int_{-\infty}^{\infty} C_x(\tau)e^{i\Omega\tau}d\tau$. The quantity Δx^2 is then defined by :

$$\Delta x^2 = \frac{1}{2\pi}\int_{-\infty}^{\infty} S_x(\Omega)d\Omega \tag{16}$$

The Langevin force spectrum is defined by :

$$S^L[\Omega] = \hbar \left| Im\frac{1}{\chi_m[\Omega]} \right| coth\frac{\hbar\Omega}{2k_BT} = \hbar M\Gamma coth\frac{\hbar\Omega}{2k_BT}\Omega \tag{17}$$

where Γ is the damping rate, χ_m is the mechanical susceptibility. This expression associates the dissipation of an oscillator (imaginary part of the mechanical susceptibility χ_m) to the spectrum of the Langevin forces, which represent the fluctuations of the system. This expression remains valid in the quantum regime delimited by the critical temperature $T_Q = \frac{\hbar\Omega_m}{k_B}$,

where $\Omega_m = 2\pi f_m$ is the angular resonant frequency of the oscillator. Submitted to Langevin forces, the position spectrum of the oscillator can be written as $S_x^T[\Omega] = |\chi_m[\Omega]|^2 S^L[\Omega]$:

$$S_x^T[\Omega] = \frac{2\Gamma}{M\left[(\Omega_m^2 - \Omega^2)^2 + \Gamma^2\Omega^2\right]}\hbar\Omega_m\left(n_T + \frac{1}{2}\right) \tag{18}$$

where $n_T = \left(e^{\frac{\hbar\Omega_m}{k_B T}} - 1\right)^{-1}$ is the Bolztmann distribution. For $T \ll T_Q$, we define the quantum fluctuation noise spectrum :

$$S_x^Q[\Omega] = \frac{\Gamma}{M\left[(\Omega_m^2 - \Omega^2)^2 + \Gamma^2\Omega^2\right]}\hbar\Omega_m \tag{19}$$

For $T \gg T_Q$, we define the thermal fluctuations spectrum of the oscillator :

$$S_x^T[\Omega] = \frac{2\Gamma}{M\left[(\Omega_m^2 - \Omega^2)^2 + \Gamma^2\Omega^2\right]}k_B T \tag{20}$$

Thus, at resonance, we obtain :

$$S_x^Q[\Omega_m] = \frac{\hbar Q}{M\Omega_m^2} \tag{21}$$

$$S_x^T[\Omega_m] = \frac{2Qk_B T}{M\Omega_m^3} \tag{22}$$

where the mechanical quality factor is defined as $Q = \frac{\Omega_m}{\Gamma}$.

When we measure the ac reflection from an thermally driven graphene membrane, we record the power spectral density (PSD) S_m of the ac output voltage delivered by the photodiode using a spectrum analyser. S_m has two contributions: i) the electrical noise S_e, and ii) the mechanical response S_z of the thermally driven membrane.

Therefore, if the oscillator is only submitted to the thermal fluctuation, we measure $S_m = S_e + S_b(P_{laser}) + (B\chi_{opt})^2 S_x^T$. The conversion factor depends on the optical susceptibility $\chi_{opt} = \frac{\partial I_r}{\partial x}$, defined as the slope of the standing wave pattern in which the membrane is moving. This interferometric response I_r is approximated by a sine function. If the initial position of the membrane is away from any extrema of I_r and in the limit of the small displacements ($\delta x \ll \lambda$), we can make the approximation : $B\chi_{opt} \sim \beta P_{laser}$. The quantity $S_b(P_{laser}) \propto P_{laser}$ represents the background noise of the laser measured by the photodetector.

First, we measure the power spectrum in units of V_{rms}. The power spectral density obtained for a given resolution bandwidth (RBW) $S_m = \frac{V_m^2}{RBW}$ thus has units of V^2Hz^{-1}.

In order to obtain the position noise S_x (in m^2Hz^{-1}), we then calculate the quantity $S_m - S_e = S_b + (\beta P_{laser})^2 S_x$. Finally, we fit the obtained signal by a function $f(\omega) =$

$\mathcal{L}(\Omega)+cte$. The constant background gives us the quantity S_b, while the Lorentzian fit $\mathcal{L}(\Omega)$ provides the mechanical frequency, Q factor and area $\mathcal{A} = \int_{-\infty}^{\infty} (\beta P_{laser})^2 S_x(\Omega) d\Omega$. In order to estimate the conversion coefficient β, we have (from Eq. 16) :

$$\int_{-\infty}^{\infty} (\beta P_{laser})^2 S_x(\Omega) d\Omega = (\beta P_{laser})^2 \frac{k_B T}{M \Omega_m^2} \tag{23}$$

From the fit we find that $\mathcal{A} = \int_{-\infty}^{\infty} (\beta P_{laser})^2 S_x(\Omega) d\Omega = 2.751\ 10^{-8}$ V^2_{rms}, and $S_b = 2.07\ 10^{-12}$ V^2_{rms} Hz^{-1}. From Eq. 23, we can evaluate the quantity :

$$\beta = \frac{\Omega_m}{P_{laser}} \sqrt{\frac{\mathcal{A} M}{k_B T}} \tag{24}$$

Thus, $\beta = 2.46 \cdot 10^{17}$V m^{-1} W^{-1} which we use to calibrate measured signal in m^2 Hz^{-1} as shown in Supplementary Figure 3 and extract the mechanical resonance at $f_m = 15.5$ MHz as well as a mechanical quality factor $Q = 156$. The background level S_b defines the sensitivity of our interferometric measurement to be on the order of 0.5 pm Hz$^{1/2}$.

Supplementary Note 3. MECHANICAL RESPONSE WITH ELECTROSTATIC DRIVE

To evaluate the oscillation amplitude x_d caused by a monochromatic electrostatic drive, we use the following expression :

$$x_d[\Omega_m] = \frac{QF}{M\Omega_m^2} = \frac{Q}{M\Omega_m^2} \partial_x C \ \left(V_g^{dc}\right) V_g^{ac} \tag{25}$$

We use the first order expression (cf. Eq. 5) of C to obtain $\partial_x C$:

$$\partial_x C = \frac{\partial}{\partial x} \left(\iint dS \frac{\epsilon_0}{\delta(x) + \frac{h}{\epsilon_r}} \right) \tag{26}$$

This leads to the simple expression :

$$|\partial_x C| = \frac{\pi R^2 \epsilon_0}{\left(\delta + \frac{h}{\epsilon_{SiO_2}}\right)^2} \tag{27}$$

Using typical values for a device with $d = \delta + \frac{h}{\epsilon_{SiO_2}} = 165$ nm, $R = 1.5$ μm we obtain $\partial_x C = 2.07$ nF m^{-1}. This yields $x_d = 3$ pm for a drive of $V_g^{dc} + V_g^{ac}$ =1 V + 100 μV and $Q = 150$.

By continuously increasing V_g^{dc}, the membrane is deflected to a point where it is in contact with the nano-diamond. Here, we describe how scanning confocal microscopy allows us to determine the value of V_g^{dc} for which contact with the nano-diamond occurs.

The position of the nano-diamond is determined by AFM scans of the device before graphene transfer, as shown in Supplementary Figure 3-a. We drive the resonator at its mechanical resonance frequency f_m with a waveform generator and detect the reflection component at this frequency with a radio-frequency lock-in amplifier. A map of the lock-in amplifier phase over the area of the oscillating resonator reveals whether the graphene touches nano-diamond or not in a straightforward manner. For instance, Supplementary Figure 3-b shows homogeneous phase over the membrane surface in its initially undeflected state at $V_g^{dc} = 0$ V. By applying an electrostatic potential $V_g^{dc} = 5$ V, we deflect the membrane to an extent where it touches the nano-diamond and ϕ_{lockin} is severely distorted around the nano-diamond location as shown in Supplementary Figure 3-c. We use this as a further method of calibration for the electrostatic deflection and the graphene-nano-diamond separation. After contact with the nano-diamond, the graphene can be detached and brought back to its initial, undeflected state by setting $V_g^{dc} = 0$ V again, as is shown in Supplementary Figure 3-d. This indicates that adhesion of a small area of the drum to the nano-diamond due to van-der-Waals forces can be overcome. The detachment of the membrane occurs on a time scale of a few hours after setting $V_g^{dc} = 0$ V.

We measure the mode profile of a given graphene resonator by driving it at one of its mechanical resonances $f_{m,i}$ (where $i = 0$ corresponds to the fundamental mode) and recording the lock-in signal at $f_{m,i}$ at each point over the resonator.

Supplementary Figure 3-e shows a finite-element simulation of the fundamental mechanical mode, while Supplementary Figure 3-f is the corresponding experimentally measured profile.

Electrostatic deflection of a graphene resonator gives rise to a tunable mechanical resonance frequency f_m, given as[15]:

$$f_m = \frac{1}{2\pi}\sqrt{\frac{4.92hE\epsilon}{m_{eff}} - \frac{0.271\epsilon_{eff}\pi R^2}{m_{eff}d^3}(V_g^{dc})^2} \qquad (28)$$

where h is the graphene sheet thickness, $E \approx 1$ TPa graphene's Young's modulus, ϵ the membrane's intrinsic strain, m_{eff} the effective mass of the mechanical mode, ϵ_{eff} the effective dielectric constant of the device and R the membrane radius.

For a graphene drum where the intrinsic strain dominates any additional strain induced by electrostatic deflection, we use Eq. 28 to describe electrostatic softening which results in decreasing f_m with V_g^{dc}.

The measurement of $f_m(V_g^{dc})$ also reveals intrinsic doping of the graphene drum, which is observed as a shift of the maximum value $f_{m,max}$ at non-zero V_0. Thus, V_0 is an offset voltage which must be taken into account to determine the deflection potential $\Delta V_g^{dc} = V_g^{dc} - V_0$ applied to a given drum.

We use Eq. 28 to fit typical detuning data shown in Supplementary Figure 3-g to find an effective mass $m_{eff} = 4.6 \cdot 10^{-17}$ kg of a four-layer graphene membrane of radius $R = 1.5\mu$m, resonating in its fundamental mode. The offset voltage is found to be $V_0 = 0.89$ V. As such, our resonator's mass density per layer is a factor 2.13 larger than graphene's intrinsic mass density $\rho_{2D} = 7.6 \cdot 10^{-19}$ kg m$^-2$, which we attribute to impurities on the membrane surface. From this, we obtain a spring constant $k = m_{eff}(2\pi f_m(0))^2 = 0.04$ N m^{-1} in the undeflected state where $V_g^{dc} = 0$ V.

Supplementary Figure 4-b shows a schematic diagram of the experimental setup used to obtain the data described in the main text. Here, our device is placed in a cryostat at 3 K and electrically connected to a voltage source which can actuate the graphene membrane on the device by a combination of DC (V_g^{dc}) and radio-frequency (δV_g^{ac}) voltages. the device is scanned with 532 nm CW laser radiation (green line) by an objective mounted on a translation stage (marked as x,y).

Reflected light (red line) is collected and split into confocal reflection and an emission channels by a dichroic mirror (transmission for $\lambda < 580$ nm). The light reflection channel is read out by splitting it with a beamsplitter (BS) and reading it out with an AC and a DC photodiode. Here, the AC signal is analysed with a spectrum analyser. A combination of a quarter-wave plate ($\frac{\lambda}{4}$), a half-wave ($\frac{\lambda}{2}$) plate and a polarizing beamsplitter (PBS) is used to control the polarization such that a maximum of reflected light is passed to the reflection channel. Similarily, the emission channel is read out by an avalanche photodiode (APD), where the combination of the dichroic mirror, two 532 nm notch filters and one longpass filter (LP) for $\lambda > 532$ nm suppress the reflection component of the signal. Finally, the APD signal is analysed by photon correlator which can count individual photons or perform a synchronous measurement triggered by the radio-frequency drive signal δV_g^{ac}.

Supplementary Note 4. NVC emission as a predominant component in the measured signal

To measure NVC emission, we use a combination of dichroic, notch and long-pass filters to suppress the excitation laser component in the reflected signal. We read out the filtered optical signal I_{APD} with an avalanche photodiode (APD). This signal depends on the position $\vec{r}$ on the device as well as the excitation laser power P. For a position $\vec{r}$ where an NVC is excited, I_{APD} consists of NVC emission I_{NVC} with an added background signal I_b:

$$I_{APD}(\vec{r}, P) = I_{NVC}(\vec{r}, P) + I_b(\vec{r}, P) \tag{29}$$

Here, I_b includes emission from the substrate, graphene and processing residues as well as a parasitic reflection component and detector noise.

A line trace of emission in Supplementary Figure 4-f shows the strong and localised increase in I_{APD} at a nano-diamond site due to NVC emission. Replacing the dichroic filter of optical density (OD) 3 with a mirror causes a net increase of I_{APD} by only 16 % at each position along the trace, indicating that reflection leaks do not contribute significantly to the measured signal. Indeed, in the case of a dominant contribution of reflection in I_{APD}, one could expect a discrepancy between the signal at the nano-diamond position and away from it as the reflected intensity should be different in each case, *e.g.* due to Rayleigh scattering from the nano-particle. This confirms that the combination of two 532 nm notch filters (each with OD 6) with the dichroic filter efficiently rejects reflected excitation light.

By measurement of I_{APD} at a position on the membrane away from an NVC, we have access to the background signal I_b. From an excitation laser power dependence of both, we extract NVC emission $I_{NVC} = I_{APD} - I_b$ as in Supplementary Figure 4-g and observe emission saturation at 30 kcps.

For a given laser power, the respective contributions of the NVC signal and background noise to the total APD signal are then given as:

$$\Delta I_{NVC} = \frac{I_{NVC}}{I_{APD}} \ ; \ \Delta I_b = \frac{I_b}{I_{APD}} \tag{30}$$

These relative contributions are presented in Supplementary Figure 4-h and show that there exists an optimum laser power $P_0 = 1$ mW at which I_{NVC} has the greatest relative contribution to I_{APD}.

At 3 K, we obtain the emission spectrum shown in Supplementary Figure 4-a from nano-diamonds containing multiple NVCs excited at 532 nm. Here, the NV^0 and NV^- transition peaks are discernible over the background of the strong phonon sideband.

In the previous section, we find the governing component of our measured signal to be NVC emission. However, multiple mechanisms may cause a modulation of this signal when the graphene membrane is deflected towards the NVC or driven at radio frequencies.

For instance, the motion of the graphene membrane may change the power absorbed in the graphene, hereby varying the power incident on the NVC and thus its emission. However, this effect can be ruled out as the measured NVC emission decreases (red curve in Supplementary Figure 4-c) with decreasing graphene-emitter separation d_{G-NVC}, while the incident power on the NVC increases (green curve in Supplementary Figure 4-c).

The measurements presented in Supplementary Figure 4-c are also well described by models of the reflection signal I_r and emission I_{APD} against d_{G-NVC} as is shown in Supplementary Figure 4-d. While I_r shows increasing and linear behaviour in the region explored by deflection of the graphene membrane (inside dashed area), I_{APD} is non-linear and diminishes for small d_{G-NVC}.

Interferometric modulation of the NVC emission itself can be neglected as the emission source is an ensemble of spatially incoherent point dipole sources with random emission direction and a broad emission spectrum (see Supplementary Figure 4-a). As such, any interference pattern produced by this source is badly defined and thus will not be modulated by the motion of the graphene membrane.

NVC emission can be modulated by electric fields due to the Stark effect, leading to a shift of NVC emission energy. Lifetime-limited linewidth NVCs (linewidth typically 10's of MHz) have shown that the Stark shift of these emitters can display linear and quadratic behaviour in the electric field strength, where the dominant contribution is typically linear and on the order of GHz/(MV m^{-1})[1,13]. In our case, NVC's have a random orientation with respect to electric field between the graphene membrane and the backgate, where the field strength is on the order of 300 MV m^{-1}. Therefore, one can expect the Stark shift to be between zero (NVC dipole oriented perpendicular to field) and some THz for a dipole oriented parallel to the field. In comparison, the emission line of the NVCs embedded in nano-diamonds is typically broad. Specifically, for the NVCs used in our present work, the emission spectrum (see Supplementary Figure 4-a) is approximately two orders of magnitude larger than the

maximum expected Stark shift.

Furthermore, we employ off-resonant excitation at 532 nm (linewidth 30 GHz), which implies that the excitation efficiency will not be affected by energy level shifts on the order of few THz. In addition, our collection is insensitive to these shifts as we collect all photons with energies smaller than our excitation energy by employing steep notch filters for the excitation line on the emission collection arm of our setup. Owing to this, we exclude the influence of the Stark effect on our measurements.

We now discuss why I_{NVC} dominates the measured signal I_{APD} when we record time-resolved emission traces. For a generic, time-varying emission signal, we determine the contribution of the emission variance σ^2_{NVC} to the total variance σ^2_{APD} of the signal, and compare it to the background variance σ^2_b:

$$\begin{aligned}\sigma^2_{APD} &= \langle I^2_{APD}\rangle - \langle I_{APD}\rangle^2 \\ &= \langle (I_{NVC}+I_b)^2\rangle - \langle (I_{NVC}+I_b)\rangle^2 \\ &= \langle I^2_{NVC}\rangle + \langle I^2_b\rangle + 2\langle I_{NVC}I_b\rangle - \langle I_{NVC}\rangle^2 - \langle I_b\rangle^2 - 2\langle I_{NVC}\rangle\langle I_b\rangle \\ &= \sigma^2_{NVC} + \sigma^2_b + 2cov\left(I_{NVC}, I_b\right)\end{aligned} \tag{31}$$

As I_{NVC} and I_b arise from independent processes, $cov\left(I_{NVC}, I_b\right) = 0$ and thus $\sigma^2_{APD} = \sigma^2_{NVC} + \sigma^2_b$.

Similarly to what has been introduced before, we now define α and β as the relative contributions of the NV emission and background signal to the total measured variance:

$$\alpha = \frac{\sigma^2_{NVC}}{\sigma^2_{NVC}+\sigma^2_b} = \frac{a^2}{a^2+b^2}\ ;\ \beta = \frac{\sigma^2_b}{\sigma^2_{NVC}+\sigma^2_b} = \frac{b^2}{a^2+b^2} \tag{32}$$

We recall that $\sigma^2(kX) = k^2\sigma(X)$, where k is a constant, and $\sigma^2(X)$ the variance of the ensemble X.

In Supplementary Figure 4-e, we compare the values of α and β. This comparison shows that NVC emission is the dominant contribution to the periodically modulated emission signal which we attribute to the NVC acting as a transducer of graphene motion by n-RET.

Energy transfer from an emitting dipole to graphene at a separation d_{G-NVC} leads to a modified total decay rate $\Gamma_G(d_{G-NVC})$ due to an additional divergent non-radiative decay channel provided by n-RET:

$$\frac{\Gamma_G(d_{G-NVC})}{\Gamma_0} = 1 + \frac{9\nu\alpha}{256\pi^3(\epsilon_r+1)^2}\left(\frac{\lambda}{d_{G-NVC}}\right)^4 \tag{33}$$

where Γ_0 is the decay rate in the absence of graphene, $\nu \in [1, 2]$ takes into account the emitting dipole orientation, α is the fine structure constant, ϵ_r is the equivalent relative permittivity of the separating medium and λ is the emission wavelength (*e.g.* 638 nm for NV^- zero phonon line). This causes emission quenching with decreasing d_{G-NVC}:

$$\frac{\Phi_G}{\Phi_0} \propto \frac{\Gamma_0}{\Gamma_G(d_{G-NVC})} \tag{34}$$

where Φ_0 is the emission in the absence of graphene.

Supplementary Note 5. CALIBRATION OF GRAPHENE-EMITTER SEPARATION USING EMISSION

To model NVC ensemble emission, we consider a nano-diamond containing $N \in [1, 4]$ NVCs emitting simultaneously. We assume an average separation of 15 nm between each NVC, located at positions $z_i \in 1, .., N$ within a nano-diamond with an average diameter of 40-60 nm. The separation of each NVC to graphene is given as $d_i = z_G - z_i$, where z_G is the position of the graphene. The ensemble emission is then given by the sum of the individual NVC emission:

$$\Phi_{ensemble}(z_G) = \Phi_{ensemble,0} \sum_N \Phi_G(d_i) \tag{35}$$

where $\Phi_{ensemble,0} = \sum_N \Phi_0$. Qualitatively, the quenching effect of an ensemble of emitters is thus strongly reduced when compared to a single NVC as is shown in Supplementary Figure 5-a.

For a single NVC near a mobile graphene membrane, Eq. 33 enables the calibration of the absolute value of the static separation d_{G-NVC} by measurement of the decay rate enhancement, e.g. by making excited state lifetime measurements by recording time-resolved emission.

However, such direct decay rate measurements cannot be used to calibrate d_i for an emitting ensemble as each NVC experiences an individual and indistinguishable decay rate enhancement. Similarly, broad-band emission measurements of an NVC ensemble using an APD cannot be used to extract information about the NVC distribution within a given nano-diamond, nor about the exact number of emitting NVCs as this information is hidden in the total emission signal.

Experimentally, we have access to the NVC emission intensity, which is related to the decay rate by Eq. 34. We measure the emission rate from the NVC at different applied backgate voltages V_g^{dc}. This data is then fitted using Eq. 34, where we substitute $d_{G-NVC} = \kappa(V_g^{dc})^2$ (cf. Eq. 10) and κ is the calibration constant. In addition, we fit our data taking into account an emission background originating from NVCs deeper within the nano-diamond and thus further away from the graphene membrane. We assume that these NVCs are not affected by the graphene and thus contribute a constant background emission signal.

Based on these assumptions, we fit our measured emission data $\Phi_{em}(V_g^{dc})$ with a simple model described by Eq. 34 which takes into account coupling by n-RET to the topmost NVC in the nano-diamond and a constant background emission signal Φ_{bg}:

$$\Phi_{em}(V_g^{dc}) = \Phi_G(V_g^{dc}) + \Phi_{bg} \tag{36}$$

We use this model to fit the measured emission for increasing V_g^{dc} in the main text, which also yields a calibration of the graphene membrane's nano-motion.

While our model shows good agreement to the measured data, it neglects n-RET coupling to other NVCs which induces a systematic error in the position detection by emission measurement. This error is enhanced by spectral broadening at 300 K, where NVC emission is broadband and thus the main emission wavelength λ is badly defined. For an optimum displacement measurement by emission, one should therefore use a system where single NVC with a narrow emission linewidth is located close to the top of the nano-diamond.

Supplementary Note 6. DERIVATION OF CALIBRATION UNCERTAINTY

We now evaluate the sources of uncertainty in the distance dependence of emission, as described by Eq. 36. This model implies an equivalent permittivity $\epsilon_{eq}(h, d, \epsilon_d)$, where d is the separation between graphene and the top surface of nano-diamond, and ϵ_d is the relative permittivity of diamond. For this serial configuration, we show that:

$$\epsilon_{eq} = \frac{(d+h)\epsilon_d}{\epsilon_d d + h} \tag{37}$$

The uncertainty on this expression is given by:

$$\Delta\epsilon_{eq} = \left| -\frac{h\epsilon_d(\epsilon_d - 1)}{(d\epsilon_d + h)^2} \right| \Delta d + \left| \frac{h(d+h)}{(d\epsilon_d + h)^2} \right| \Delta\epsilon_d + \left| \frac{d\epsilon_d^2}{(d\epsilon_d + h)^2} \right| \Delta h \tag{38}$$

For $d = 65 \pm 10$ nm, $\epsilon_d = 7 \pm 2$ and $h = 5 \pm 5$ nm, we obtain $\epsilon_{eq} = 1.07 \pm 0.06$, corresponding to an relative uncertainty of 5.6% on ϵ_{eq}. We now consider the equation governing the emission, taking into account the precedent hypothesis:

$$\Phi_G = \frac{\Phi_0}{1 + \frac{9\nu\alpha}{256\pi^3(\epsilon_{eq}+1)^2}\left(\frac{\lambda}{z}\right)^4} + \Phi_{bg} \tag{39}$$

where $z = d + h$ is the separation between the graphene and the NVC. The parameter dependence on this expression is given by:

$$d\Phi_G = \frac{\partial \Phi_G}{\partial \Phi_0} d\Phi_0 + \frac{\partial \Phi_G}{\partial \epsilon_{eq}} d\epsilon_{eq} + \frac{\partial \Phi_G}{\partial \lambda} d\lambda + \frac{\partial \Phi_G}{\partial z} dz + \frac{\partial \Phi_G}{\partial \Phi_{bg}} d\Phi_{bg} \tag{40}$$

$$\begin{aligned}
\Delta\Phi_G = & \left|\left(1 + \frac{9\nu\alpha}{256\pi^3(\epsilon_{eq}+1)^2}\left(\frac{\lambda}{z}\right)^4\right)^{-1}\right| \Delta\Phi_0 \\
& + \left|-\Phi_0\left(1 + \frac{9\nu\alpha}{256\pi^3(\epsilon_{eq}+1)^2}\left(\frac{\lambda}{z}\right)^4\right)^{-2}\left[\frac{-9\nu\alpha}{128\pi^3(\epsilon_{eq}+1)^3}\left(\frac{\lambda}{z}\right)^4\right]\right| \Delta\epsilon_{eq} \\
& + \left|-\Phi_0\left(1 + \frac{9\nu\alpha}{256\pi^3(\epsilon_{eq}+1)^2}\left(\frac{\lambda}{z}\right)^4\right)^{-2}\left[\frac{9\nu\alpha\lambda^3}{64\pi^3(\epsilon_{eq}+1)^2 z^4}\right]\right| \Delta\lambda \\
& + \left|-\Phi_0\left(1 + \frac{9\nu\alpha}{256\pi^3(\epsilon_{eq}+1)^2}\left(\frac{\lambda}{z}\right)^4\right)^{-2}\left[\frac{-9\nu\alpha\lambda^4}{64\pi^3(\epsilon_{eq}+1)^2 z^5}\right]\right| \Delta z \\
& + \Delta\Phi_{bg}
\end{aligned} \tag{41}$$

As we do not have direct access to z, we introduce a transduction factor ζ such as $z = d - \zeta\left(V_g^{dc}\right)^2$. We show that :

$$\Delta z = \Delta d + \left|-2\zeta V_g^{dc}\right| \Delta\zeta + \left|\left(V_g^{dc}\right)^2\right| \Delta V_g^{dc} \tag{42}$$

Note that the uncertainty depends on the membrane position, and should be calculated at every z. Interestingly, the calculation leads to the extraction of the two dominant factors for $\Delta\Phi_G$: the uncertainty on the emission wavelength and on the background from deeply implanted NVCs. It is worth noting that the emission wavelength is intrinsically poorly defined at high temperature as the NVC spectrum is rather broad, while it becomes narrower at cryogenic temperatures.

Similarly, the differential emission is given by:

$$\frac{\partial \Phi_G}{\partial z} = \frac{4\Phi_0 \frac{9\nu\alpha}{256\pi^3(\epsilon_{eq}+1)^2}\frac{\lambda^4}{z^5}}{\left[1 + \frac{9\nu\alpha}{256\pi^3(\epsilon_{eq}+1)^2}\left(\frac{\lambda}{z}\right)^4\right]^2} \tag{43}$$

The parameter dependence on this expression is given by:

$$d\left(\frac{\partial \Phi_G}{\partial z}\right) = \frac{\partial \Phi_G^2}{\partial z \partial \Phi_0} d\Phi_0 + \frac{\partial \Phi_G^2}{\partial z \partial \epsilon_{eq}} d\epsilon_{eq} + \frac{\partial \Phi_G^2}{\partial z \partial \lambda} d\lambda + \frac{\partial \Phi_G^2}{\partial^2 z} dz + \frac{\partial \Phi_G^2}{\partial z \partial \Phi_{bg}} d\Phi_{bg} \tag{44}$$

The calculation of each partial derivative is simple if we use the Schwartz theorem, with which one can compute a numerical value for each error.

Supplementary Note 7. EXTRACTION OF DISSIPATIVE OPTOMECHANICAL COUPLING STRENGTH

To extract a dissipative optomechanical coupling rate, we define the following variables:

- $\Gamma_0 = 30MHz$, the intrinsic radiative decay rate of NV centres in a nanodiamond[11].
- $\Gamma_G\left(d_{G-NVC}\right)$, the non-radiative decay rate due to n-RET from the NVC to graphene.
- N_e the population in the excited state.
- $\Gamma_{tot}(z) = \Gamma_0 + \Gamma_G(z)$ the total decay rate. Doing a lifetime measurement of our device, we can extract a mean value of $\Gamma_{tot} = 100MHz$ for typical NVC located below a graphene NEMS. A representative measurement is shown in figure 5-b.
- $\Phi = \frac{\Gamma_0}{\Gamma_{tot}}\left(\Gamma_{tot}N_e\right) = N_e\Gamma_0$, the number of emitted photons per second [Hz] as a function of graphene-NVC spacing $d_{G-NVC} := z$. The quantity $\frac{\Gamma_0}{\Gamma_{tot}}$ is a branching ratio for radiative decay. $\Gamma_{tot}N_e$ is the total rate of excited population decay. The quantity Φ is proportional to the measured emission shown in Figure 2 of the main manuscript.

Following the procedure of Wu et al.[17], the detection sensitivity of our readout scheme is $\frac{d\Phi}{dz} = \frac{d\Phi}{d\Gamma_G}\frac{d\Gamma_G}{dz}$. Here, $\frac{d\Gamma_G}{dz}$ is a dissipative optomechanical coupling rate used in the formalism[6] of Elste et al. From experimental data shown in Figure 2 of the main text, the highest detection sensitivity for the device shown is $\frac{d\Gamma_G}{dz} = 300Hz.nm^{-1}$ at $z = 30nm$. One would then evaluate $|\frac{d\Phi}{d\Gamma_G}| = \frac{\Phi}{\Gamma_{tot}} = 5\ 10^{-5}$, using the background-corrected experimental value for Φ. Together with the experimental value for $\frac{d\Gamma_G}{dz}$, this yields $\frac{d\Gamma_G}{dz} = 6\ 10^6 Hz.nm^{-1}$. Given a zero-point motion amplitude $z_{ZPM} = 65fm$, we thus extract the dimensionless coupling strength[6]:

$$\tilde{B} = \frac{d\Gamma_G}{dz}\ z_{ZPM}\Gamma_0^{-1} = 1.3\ 10^{-5} \tag{45}$$

This formalism allows a quantitative comparison of dispersive and dissipative coupling. In the present study, it is clear that $\tilde{B} \ll 1$, thus our system operates far from the ultrastrong coupling regime where $\tilde{B} > 1, \gamma_M/\Gamma_0$. Nonetheless, the value of $\tilde{B} \sim 10^{-5}$ achieved by our system compares favourably to other optomechanical systems such as a microdisk coupled to an optical waveguide[10] ($\tilde{B} = 3\ 10^{-7}$), a graphene resonator coupled to a microsphere optical resonator[5] ($\tilde{B} = 8.3\ 10^{-7}$) or a photonic crystal nanocavity[17] ($\tilde{B} = 9.7\ 10^{-6}$). The

potential of dissipative optomechanical coupling for the manipulation of a mechanical degree of freedom is promising[16] as it may allow optomechanical cooling to the mechanical ground state while alleviating the "good cavity" limit $\Gamma_0 \ll \Omega_m$, which remains a major hurdle for nanoscale optomechanics

SUPPLEMENTARY REFERENCES

---

1 L. C. Bassett, F. J. Heremans, C. G. Yale, B. B. Buckley, and D. D. Awschalom. Electrical tuning of single nitrogen-vacancy center optical transitions enhanced by photoinduced fields. *Physical Review Letters*, 107(26), 2011.

2 P. Blake, E. W. Hill, A. H. Castro Neto, K. S. Novoselov, D. Jiang, R. Yang, T. J. Booth, and A. K. Geim. Making graphene visible. *Applied Physics Letters*, 91(6):063124, 2007.

3 Andreas Brenneis, Louis Gaudreau, Max Seifert, Helmut Karl, Martin S Brandt, Hans Huebl, Jose a Garrido, Frank H L Koppens, and Alexander W Holleitner. Ultrafast electronic read-out of diamond NV centers coupled to graphene. *Nature nanotechnology*, 10(2):135–139, 2014.

4 George F. Burkhard, Eric T. Hoke, and Michael D. McGehee. Accounting for interference, scattering, and electrode absorption to make accurate internal quantum efficiency measurements in organic and other thin solar cells. *Advanced Materials*, 22:3293–3297, 2010.

5 Robin M. Cole, George a. Brawley, Vivekananda P. Adiga, Roberto De Alba, Jeevak M. Parpia, Bojan Ilic, Harold G. Craighead, and Warwick P. Bowen. Evanescent-Field Optical Readout of Graphene Mechanical Motion at Room Temperature. *Physical Review Applied*, 3:1–7, 2015.

6 Florian Elste, S. M. Girvin, and a. a. Clerk. Quantum noise interference and backaction cooling in cavity nanomechanics. *Physical Review Letters*, 102(May):1–5, 2009.

7 a. C. Ferrari, J. C. Meyer, V. Scardaci, C. Casiraghi, M. Lazzeri, F. Mauri, S. Piscanec, D. Jiang, K. S. Novoselov, S. Roth, and a. K. Geim. Raman spectrum of graphene and graphene layers. *Physical Review Letters*, 97(18):187401, 2006.

8 Mikhail Katsnelson. *Graphene : carbon in two dimensions.* Cambridge University Press, 2012.

9 Landau L, Lifshitz E, Sykes J, Reid W, and Dill E. *Theory of elasticity: Vol. 7 of course of theoretical physics.* Pergamon Press, 1970.

[10] Mo Li, Wolfram H P Pernice, and Hong X Tang. Reactive Cavity Optical Force on Microdisk-Coupled Nanomechanical Beam Waveguides. *Physical Review Letters*, 103(22):223901, 2009.

[11] A. Mohtashami and A. F. Koenderink. Suitability of nanodiamond nitrogen-vacancy centers for spontaneous emission control experiments. *New Journal of Physics*, 15:043017, 2013.

[12] R R Nair, P Blake, A N Grigorenko, K S Novoselov, T J Booth, T Stauber, N M R Peres, and A K Geim. Fine structure constant defines visual transparency of graphene. *Science (New York, N.Y.)*, 320(5881):1308, June 2008.

[13] Ph Tamarat, T. Gaebel, J. R. Rabeau, M. Khan, A. D. Greentree, H. Wilson, L. C L Hollenberg, S. Prawer, P. Hemmer, F. Jelezko, and J. Wrachtrup. Stark shift control of single optical centers in diamond. *Physical Review Letters*, 97(8):083002, August 2006.

[14] Julia Tisler, Thomas Oeckinghaus, Rainer J Stöhr, Roman Kolesov, Rolf Reuter, Friedemann Reinhard, and Jörg Wrachtrup. Single defect center scanning near-field optical microscopy on graphene. *Nano letters*, 13(7):3152–6, July 2013.

[15] P Weber, J Güttinger, I Tsioutsios, D E Chang, and a Bachtold. Coupling graphene mechanical resonators to superconducting microwave cavities. *Nano letters*, 14(5):2854–60, May 2014.

[16] Talitha Weiss, Christoph Bruder, and Andreas Nunnenkamp. Strong-coupling effects in dissipatively coupled optomechanical systems. *New Journal of Physics*, 15(4):045017, apr 2013.

[17] Marcelo Wu, Aaron C Hryciw, Chris Healey, David P Lake, Harishankar Jayakumar, Mark R Freeman, John P Davis, and Paul E Barclay. Dissipative and Dispersive Optomechanics in a Nanocavity Torque Sensor. *Physical Review X*, 4(2):21052, 2014.
